# A Role of Corazonin Receptor in Larval-Pupal Transition and Pupariation in the Oriental Fruit Fly *Bactrocera dorsalis* (Hendel) (Diptera: Tephritidae)

**DOI:** 10.3389/fphys.2017.00077

**Published:** 2017-02-15

**Authors:** Qiu-Li Hou, Hong-Bo Jiang, Shun-Hua Gui, Er-Hu Chen, Dan-Dan Wei, Hui-Min Li, Jin-Jun Wang, Guy Smagghe

**Affiliations:** ^1^Key Laboratory of Entomology and Pest Control Engineering, College of Plant Protection, Southwest UniversityChongqing, China; ^2^Department of Crop Protection, Ghent UniversityGhent, Belgium

**Keywords:** neuropeptide, corazonin, corazonin receptor, *Bactrocera dorsalis*, expression pattern, RNA interference, larval-pupal transition, pupariation behavior

## Abstract

Corazonin (Crz) is a neuropeptide hormone, but also a neuropeptide modulator that is internally released within the CNS, and it has a widespread distribution in insects with diverse physiological functions. Here, we identified and cloned the cDNAs of *Bactrocera dorsalis* that encode Crz and its receptor CrzR. Mature *BdCrz* has 11 residues with a unique Ser^11^ substitution (instead of the typical Asn) and a His in the evolutionary variable position 7. The *BdCrzR* cDNA encodes a putative protein of 608 amino acids with 7 putative transmembrane domains, typical for the structure of G-protein-coupled receptors. When expressed in Chinese hamster ovary (CHO) cells, the *BdCrzR* exhibited a high sensitivity and selectivity for Crz (EC_50_ ≈ 52.5 nM). With qPCR, the developmental stage and tissue-specific expression profiles in *B. dorsalis* demonstrated that both *BdCrz* and *BdCrzR* were highly expressed in the larval stage, and *BdCrzR* peaked in 2-day-old 3rd-instar larvae, suggesting that the *BdCrzR* may play an important role in the larval-pupal transition behavior. Immunochemical localization confirmed the production of Crz in the central nervous system (CNS), specifically by a group of three neurons in the dorso-lateral protocerebrum and eight pairs of lateral neurons in the ventral nerve cord. qPCR analysis located the *BdCrzR* in both the CNS and epitracheal gland, containing the Inka cells. Importantly, dsRNA-*BdCrzR*-mediated gene-silencing caused a delay in larval-pupal transition and pupariation, and this phenomenon agreed with a delayed expression of tyrosine hydroxylase and dopa-decarboxylase genes. We speculate that CrzR-silencing blocked dopamine synthesis, resulting in the inhibition of pupariation and cuticular melanization. Finally, injection of Crz in head-ligated larvae could rescue the effects. These findings provide a new insight into the roles of Crz signaling pathway components in *B. dorsalis* and support an important role of CrzR in larval-pupal transition and pupariation behavior.

## Introduction

Neuropeptides play a central role in regulating numerous vital physiological systems and behavioral events in diverse insects. The multiple functions of insect neuropeptides make them a prime practical potential target in the development of novel insect control agents (Boonen et al., 2009). Corazonin (Crz) is an amidated undecapeptide, which originally was isolated as a cardioacceleratory peptide from the cockroach *Periplaneta americana* (Veenstra, 1989). A closely related peptide [His^7^]-Crz was identified as a dark-inducing peptide in the locust *Schistocerca americana* (Veenstra, 1991). Two major members of the Crz family are [Arg^7^]-Crz and [His^7^]-Crz, while other variants of Crz have also been identified from a variety of insects and crustaceans (Predel et al., 1994, 2007; Veenstra, 2009; Sha et al., 2012; Sugahara et al., 2015); good examples are [Thr^4^, His^7^]-Crz in the honey bee *Apis mellifera* (Roller et al., 2006), [Gln^10^]-Crz in the kissing bug *Rhodnius prolixus*, [His^4^, Gln^7^]-Crz in members of the order Mantophasmatodea, [Tyr^3^, Gln^7^, Gln^10^]-Crz in the bumble bee (Predel et al., 2007; Sha et al., 2012), and a very unusual [Met^2^, Arg^10^]-Crz has recently be predicted from the bed bug genome (Benoit et al., 2016). Crz is primarily produced by a major group of neurosecretory cells in the pars lateralis and ventral nerve cord (VNC) within the central nervous system (CNS) and then released into the hemocoel by the corpora cardiaca (Veenstra and Davis, 1993; Cantera et al., 1994; Choi et al., 2005; Lee et al., 2008; Veenstra, 2009). These findings demonstrate a widespread distribution in diverse insect groups.

Crz is highly conserved with respect to spatial expression pattern, but no clear pattern of function has emerged. For example, the myotropic activities of Crz signaling systems have been demonstrated in the cockroach *P. americana* (Veenstra, 1989) and the stick insect *Carausius morosus* (Predel et al., 1999). An injection of Crz into *Bombyx mori* larvae reduced the silk spinning rate and prolonged pupal development, which indicates that potential functions can be associated with molting and behavior (Tanaka et al., 2002). This peptide was shown to be involved in the initiation of ecdysis behavior by injection of Crz into pharate larvae in the moth *Manduca sexta* (Kim et al., 2004). In the desert locusts *Locusta migratoria* and *Schistocerca gregaria* knockdown of the Crz gene revealed its critical role in the melanization pattern and phase polyphenism (Tanaka, 2000b; Sugahara et al., 2015). Furthermore, it has been reported that the ecdysis-triggering hormone (ETH) from the endocrine Inka cells initiates the ecdysis process through a direct action on the CNS (Park et al., 2002a). Subsequently, there is an upregulation of the genes of tyrosine hydroxylase (TH) and dopa-decarboxylase (DDC) that is required for a successful cuticle tanning/sclerotization after the ecdysis process (Huang et al., 2005; Gorman and Arakane, 2010). Crz-producing neurons in *Drosophila melanogaster* involve regulation of trehalose levels (Lee et al., 2008), and Crz receptor (CrzR) knockdown affects starvation resistance and modulates stress responses and metabolism (Veenstra, 2009; Boerjan et al., 2010; Zhao et al., 2010; Kubrak et al., 2016). Of surprise, the Crz signaling pathway is considered to be absent in tenebrionid beetles and aphids because the peptide and its cognate receptors were not found, and its biological activity and immunoreactivity were undetectable in any of these species examined (Tanaka, 2000a; Sugahara et al., 2015).

Signaling via G protein-coupled receptors (GPCRs) is a major route of cellular communication via the plasma membrane. Identification of the Crz receptor gene of *D. melanogaster* (*DmCrzR*) was the first step in assigning unrecognized functions for this peptide (Cazzamali et al., 2002; Park et al., 2002b). To investigate the role of the Crz signaling pathway, we isolated and characterized the *DmCrz* and *DmCrzR* ortholog from the oriental fruit fly, *Bactrocera dorsalis* (Hendel) (Diptera: Tephritidae). *B. dorsalis*, a quarantine polyphagous pest, is economically one of the most important pests of tropical and subtropical regions of the world (And and Foote, 1960). Much damage of the fruit this pest inflicts occurs through oviposition punctures and subsequent larval development (Fletcher, 1987). Its wide distribution, invasiveness and potential influence on food production suggest that *B. dorsalis* is a threat to fruit industries in many countries. Moreover, due to its rapid development of insecticide resistance, the control of this pest has become more and more difficult (Chen et al., 2013). Therefore, it is urgent to find new insecticidal targets, and we believe our efforts in clarifying the functions of Crz signaling pathway in *B. dorsalis* are important and may provide new alternatives for pest control (Altstein, 2001; Nässel and Winther, 2010).

In the present study, we aimed to (1) identify and characterize the full-length cDNAs of Crz and its cognate receptor in *B. dorsalis* (*BdCrz* and *BdCrzR*), (2) demonstrate the sensitivity and selectivity of the *BdCrzR* for Crz mature peptides, (3) analyze the spatial and temporal expression patterns of *BdCrz* and *BdCrzR*, and (4) elucidate the function of *BdCrzR* by RNAi, with specific interest on the larval-pupal transition and pupariation behavior. This study will contribute to a better understanding of the roles of the Crz signaling components in *B. dorsalis* and also shed light on their potential as the target of novel insect control agents.

## Materials and methods

### Insects and chemicals

The laboratory colony of *B. dorsalis* was originally collected in 2008 from Dongguan, Guangdong province, China. Adult flies are reared in plastic cages (30 × 30 × 40 cm) under laboratory condition at 27 ± 1°C, 70 ± 5% relative humidity with a photoperiod of 14 h light: 10 h dark. The different developmental stages of flies were synchronized and collected as described previously (Chen et al., 2013). Briefly, females were allowed to oviposit into pinpricked plastic tubes (50 mL) containing fresh orange pulp, and the eggs were collected. After hatching, the larvae were fed on an artificial diet consisting of yeast powder, sucrose, corn and wheat flour, and later the 3rd-instar larvae were transferred into a plastic basin containing sand for pupation. Pupae were sieved from the sand and placed in plastic cages with adult food (sucrose:yeast hydrolysate = 3:1) and water.

The peptides of Crz and ETH of *B. dorsalis* were synthesized by Genescript (Nanjing, China), and the adipokinetic hormone (AKH) peptide of *Bombyx mori* (*BmAKH*) was a gift from Dr. Naiming Zhou (Zhejing University, China; Yang et al., 2013). The amino acid (aa) sequences for Crz, ETH and AKH were added in Table 1. Plasmids were prepared using the MIDIprep kit as purchased from Qiagen (Valencia, CA). The reagents for cell culture and GPCR assay were purchased from Gibco cell culture at Life Technologies (Grand Island, NY), including fetal bovine serum, DMEM/F12 medium, fungizone, penicillin/streptomycin, and coelenterazine. The transfection reagent (TransIt) was purchased from Mirus Bio (Madison, WI).

**Table 1:** **List of peptides used for the activity relationship studies and recuse assay**.

**Peptide**	**Sequence**
*BdCrz*	pGln-Thr-Phe-Gln-Tyr-Ser-His-Gly-Trp-Thr-Ser-NH2
*BmAKH*	pCys-Glu-Leu-Thr-Phe-Ser-Pro-Asp-Thr-NH2
*BdETH*	pAsn-Glu-Ser-Pro-Gly-Phe-Phe-Leu-Lys-Ile-Thr-Lys-Asn-Val-Pro-Arg-Leu-NH2

### Sample preparation of developmental stages and tissues

The developmental stage-specific expression profiles of *B. dorsalis* were established using samples of eggs, larvae (1-, 4-, 7-day-old), pupae (1-, 4-, 7-day-old), and adults (1-, 5-, 9-day-old). Five randomly collected insects were pooled as one sample for stage-specific expression profiling, with three independent biological replications per stage. Tissue from the CNS (both brain and ventral nerve cord), gut (the complete digestive tract), fat body, Malpighian tubules, epidermis, and the epitracheal gland (EG), containing the Inka cells, were excised from 2-day-old 3rd-instar larvae (i.e., 7-day-old larvae; this is the moment prior to larval-pupal transition) to determine the tissue-specific expression patterns. At least 15 individuals were dissected as one sample for tissue-specific analysis, and three independent biological replications were done per tissue. The larvae were chilled on ice for 30 min and dissected under a stereomicroscope (Olympus SZX12, Tokyo, Japan). The samples were isolated on ice, placed in a 2.0 mL-diethyl pyrocarbonate-treated centrifuge tube containing RNA storage reagent (Tiangen, Beijing, China), and immediately frozen in liquid nitrogen and stored at −80°C.

### RNA isolation and cDNA synthesis

Total RNA was extracted from each sample using the TRIzol reagent (Invitrogen, Carlsbad, CA) and treated with DNase I (Promega, Madison, WI) to remove contaminating genomic DNA. First-strand cDNA was synthesized using the GoScript Reverse Transcription System (Promega) with random hexamer primers and oligo (dT) in a total volume of 20 μL, according to the manufacturer's instructions.

### Identification and sequencing of *BdCrz* and *BdCrzR* cDNAs

Based on the *B. dorsalis* genome database (https://i5k.nal.usda.gov/Bactrocera_dorsalis), the *BdCrz* and *BdCrzR* genes were identified by performing a TBLASTN search using the Crz and CrzR homologs of *D. melanogaster* (Veenstra, 1994; Cazzamali et al., 2002). The identities of recovered cDNAs of *B. dorsalis* were confirmed by BLASTx analyses. The full open reading frame (ORF) of *BdCrz* and *BdCrzR* were amplified utilizing nested PCR using the high fidelity DNA polymerase PrimeSTAR (Takara, Dalian, China). The primers (Table S1) were designed based on *B. dorsalis* genome data. The PCR conditions were: initial denaturation at 95°C for 2 min, followed by 32 cycles of 30 s at 95°C, 15 s at 55°C, and 2 min at 72°C, and final extension of 5 min at 72°C. The 50 μL-PCR mixture included 24 μL of ultrapure water, 20 μL of 2 × PrimeSTAR Max Premix (TaKaRa), 2 μL of each primer (10 μM) and 2 μL of the cDNA template. The PCR products were separated by agarose gel electrophoresis and purified by DNA extraction kit (TaKaRa). The purified PCR products were subcloned into the pGEM-T Easy vector (Promega) and then sequenced (BGI, Shenzhen, China).

### Sequence and phylogenetic analysis

The signal peptide of the peptide precursor was predicted using SignalP Server (http://www.cbs.dtu.dk/services/SignalP). The transmembrane domains of the GPCR were predicted using TMHMM server (http://www.cbs.dtu.dk/services/TMHMM). All sequences were aligned using the Clustal X2 software (Larkin et al., 2007) with default settings. The AKH receptor of *D. melanogaster* (*DmAKHR*) was included as outgroup. The neighbor-joining (NJ) tree was produced in MEGA 6.0 (Tamura et al., 2013) with 1000 bootstrap replicates.

### Quantitative real-time polymerase chain reaction (qPCR)

qPCR was performed in 10 μL-reaction mixtures containing 5 μL of GoTaq qPCR Master Mix (Promega), 0.5 μL of cDNA template, 3.5 μL of ddH_2_O and 0.5 μL of each primer (0.2 mM). The gene-specific qPCR primers are presented in Table S1. qPCR was performed on an ABI 7500 Real-Time PCR System (Applied Biosystems, Foster City, CA) under the following reaction conditions: an initial denaturation at 95°C for 2 min, followed by 40 cycles of 95°C for 15 s, 60°C for 30 s. At the end of each qPCR, a melting curve analysis from 60 to 95°C was generated to rule out the possibility of primer-dimer formation. The data were normalized to the stable reference gene α-*tubulin* (GenBank accession no. GU269902) based on previous evaluations (Shen et al., 2010). The relative expression levels were calculated using the 2^−ΔΔCt^ method (Livak and Schmittgen, 2001).

### Heterologous expression and Ca^2+^ luminescence assay

The ORF for *BdCrzR* was inserted into the expression vector pcDNA3.1 (+). The correct clone of pcDNA3.1-*BdCrzR* was confirmed by sequencing (Invitrogen). Chinese Hamster Ovary (CHO) cells, supplemented with aequorin, and Gα16 subunit, were employed for the heterologous expression. The methods for the transfection and the Ca^2+^ mobilization assay were performed as previously described (Jiang et al., 2014). Briefly, cells were transfected with the pcDNA3.1(+)-*BdCrzR* using TransIT-LT1 transfection reagent (Mirus Bio). At 30 h after the transfection, the cells were collected and incubated with the coelenterazine (Invitrogen) for 2.5 h prior to the functional assay. The cells transfected with the empty pcDNA3.1 vector, were used as a negative control.

The luminescence assay was performed by measuring the intracellular Ca^2+^ mobilization of the transfected CHO cells. A 10-fold serial dilution of the peptides (*BdCrz* and *BmAKH*) was applied to the cells. The luminescence caused by the intracellular calcium mobilization was measured within 20 s in every half-second intervals by a TriStar^2^ LB 942 Multimode Reader (Berthold Technologies, Bad Wildbad, Germany; Jiang et al., 2013, 2014). The lowest concentration of *BdCrz* ligand showing the maximum activation of the receptor, was selected for luminescence normalization. Based on that, a concentration-response curve was calculated for *BdCrz* by logistic fitting in Origin 8.6 (OriginLab Corporation, Northampton, MA). All experiments were conducted with three biological replicates.

### Immunohistochemistry

The rabbit antibody against Crz (QTFQYSRGWTNamide) was a gift from Dr. Jan Veenstra at Université de Bordeaux, France (Veenstra, 1991). For immunohistochemistry, the brains from 2-day-old 3rd-instar larvae were dissected in chilled PBS (pH 7.4) and then tissues were fixed overnight at 4°C in fresh 4% paraformaldehyde in PBS. After washing for 3 × 5 min in PBS with 0.5% Triton X-100 (PBST), the tissues were incubated with primary antibody (1:1000 diluted in PBST) for 2 days at 4°C and then washed in 3 × 5 min in PBST. Tissues were incubated overnight at 4°C in Alexa 488-conjugated goat anti-rabbit IgG antibody (1:1000 in PBST). The samples were washed in 2 × 5 min in PBST. Then, samples were mounted on a clean slide with 100% glycerol. Images were captured in a confocal microscope Zeiss LSM780 (Zeiss, Jena, Germany).

### RNAi and pupariation analysis

Primers (Table S1) with T7 promoter sequences were used to amplify a fragment of *BdCrzR* or *GFP* (CAA58789) for the double-stranded RNA (dsRNA) synthesis. The dsRNA was transcribed by Transcript Aid T7 High Yield Transcription Kit (Thermo Scientific, Lithuania) following the manufacturer's protocol. The dsRNAs were quantified using a NanoPhotometer (Implen GmbH, Germany) and their integrity confirmed by a 1% agarose gel electrophoresis.

For the RNAi experiments, 2-day-old 3rd-instar larvae (i.e., 7-day-old larvae) were used, which is the moment prior to larval-pupal transition. Per larva, 300 nL of dsRNA (1.2 μg) (dsCrzR or dsGFP) or PBS was injected at the junction between the 2nd and 3rd abdominal segment using a Nanoject II Auto-Nanoliter Injector (Drummond Scientific, Broomall, PA). Then injected larvae were kept on an artificial diet under the conditions described above, and we followed morphological changes and the pupal development and weight. The level of gene-silencing of CrzR was followed by qPCR as described above. In an extra parallel experiment, we also injected dsCrzR a 2nd and 3rd time at 24 h intervals to investigate permanent gene-silencing of Crz expression and continued delay of pupal development. For all RNAi experiments, we used 20 larvae per treatment and three biological replicates were conducted.

### Rescue by peptide injection in head-ligated larvae

The experimental setup with head-ligated 2-day-old 3rd-instar larvae was based on a previous study (Kim et al., 2004). We selected the larvae and head-ligated these between the first three segments, so to cut-off the brain neurosecretory cells (NSCs) producing Crz, and followed these for pupal development that normally happens after 24 h as seen in non-ligated individuals. Upon head-ligation, a series of larvae was injected with Crz (0.3 pmol in 300 nL) or ETH (0.3 pmol in 300 nL). Upon injection larvae were kept on an artificial diet as described above, and we scored pupal development at 24 h post-injection. Per treatment, we used 20 larvae and three biological replicates were conducted. Typical phenotype effects were captured with a Leica M205A stereomicroscope with a camera (Leica Microsystems, Wetzlar, Germany).

### Statistical analysis

The data of single injection assay was all analyzed using one-way ANOVA, and significant differences between means were tested with Duncan's Multiple Range Test (*P* = 0.05) using SPSS 16.0 software (SPSS Inc., Chicago, IL, 2008). The data of the double and triple injection assay, and of the gene expression levels of ETH, TH, and DDC were analyzed using an independent Student's *t*-test in SPSS 16.0 software. Means ± SE (standard error) are based on three biological replications. Statistical significance was assumed for *p* < 0.05.

## Results

### Characterization of cDNA coding for the *BdCrz* and *BdCrzR*

**Figure 1:**
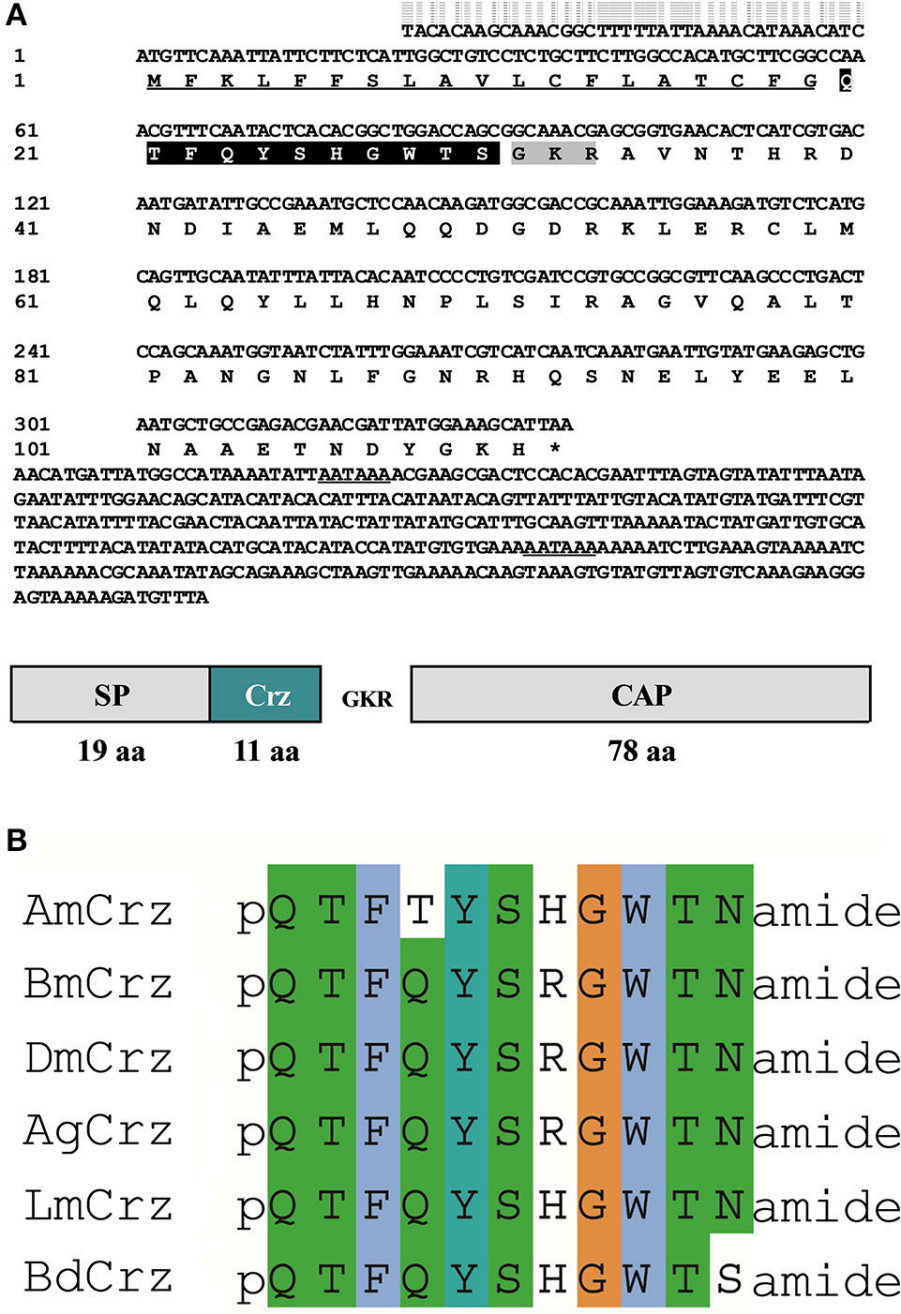
**Crz precursor in ***B. dorsalis***. (A)** The cDNA and deduced aa sequence of *BdCrz* precursor. The underlined sequence indicates signal peptide (SP); highlighted in black represents mature peptide; shaded in gray is the putative cleavage site, followed by Crz-associated peptide (CAP). The putative polyadenylation signal in the 3′-noncoding region is underlined. **(B)** Alignment of aa sequence of Crz mature peptide in different insects. Corazonin of *Apis mellifera* (*AmCrz*, AFE02890.1), *Bombyx mori* (*BmCrz*, BAC66443.1), *Drosophila melanogaster* (*DmCrz*, NP_524350.1), *Anopheles gambiae* (*AgCrz*, AFE02890.1), and *Locusta migratoria* (*LmCrz*, AKN21243.1) were used for alignment.

Full length cDNA clones encoding Crz and CrzR were isolated from *B. dorsalis* by nested PCR methods, and named *BdCrz* and *BdCrzR*, respectively. The cDNA of *BdCrz* is 740 bp long (GenBank accession no. KX831393), including an ORF of 336 bp that encodes an 111 amino acids (aa) protein (Figure 1A). The peptide consists of a 19 aa-predicted N-terminal signal peptide (SP), followed by an 11 aa-conserved mature Crz peptide “QTFQYSHGWTSamide,” and then by a 78 aa-*Crz*-associated peptide (CAP). Between the mature peptide and the CAP is a flanking dibasic cleavage site KR. In addition, a canonical amidation site G at its C-terminal end suggests that *BdCrz* is an amidated peptide. The alignment presents consensus aa sequences of the Crz mature peptides in different insects (Figure 1B).

**Figure 2:**
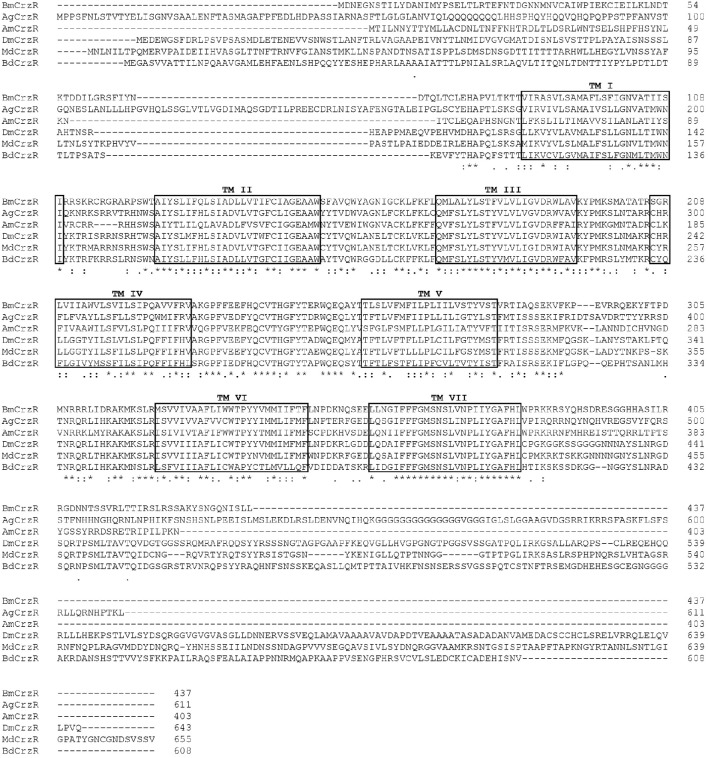
**Amino acid sequence alignment of the ***BdCrzR*** with other related GPCRs**. Conservative aa are indicated by asterisks. Seven transmembrane domains are highlighted in black box (TM I–TM VII). The corazonin receptor of *Bombyx mori* (*BmCrzR*), *Anopheles gambiae* (*AgCrzR*), *Apis mellifera* (*AgCrzR*), *Drosophila melanogaster* (*DmCrzR*), and *Musca domestica* (*MdCrzR*) were used for alignment.

**Figure 3:**
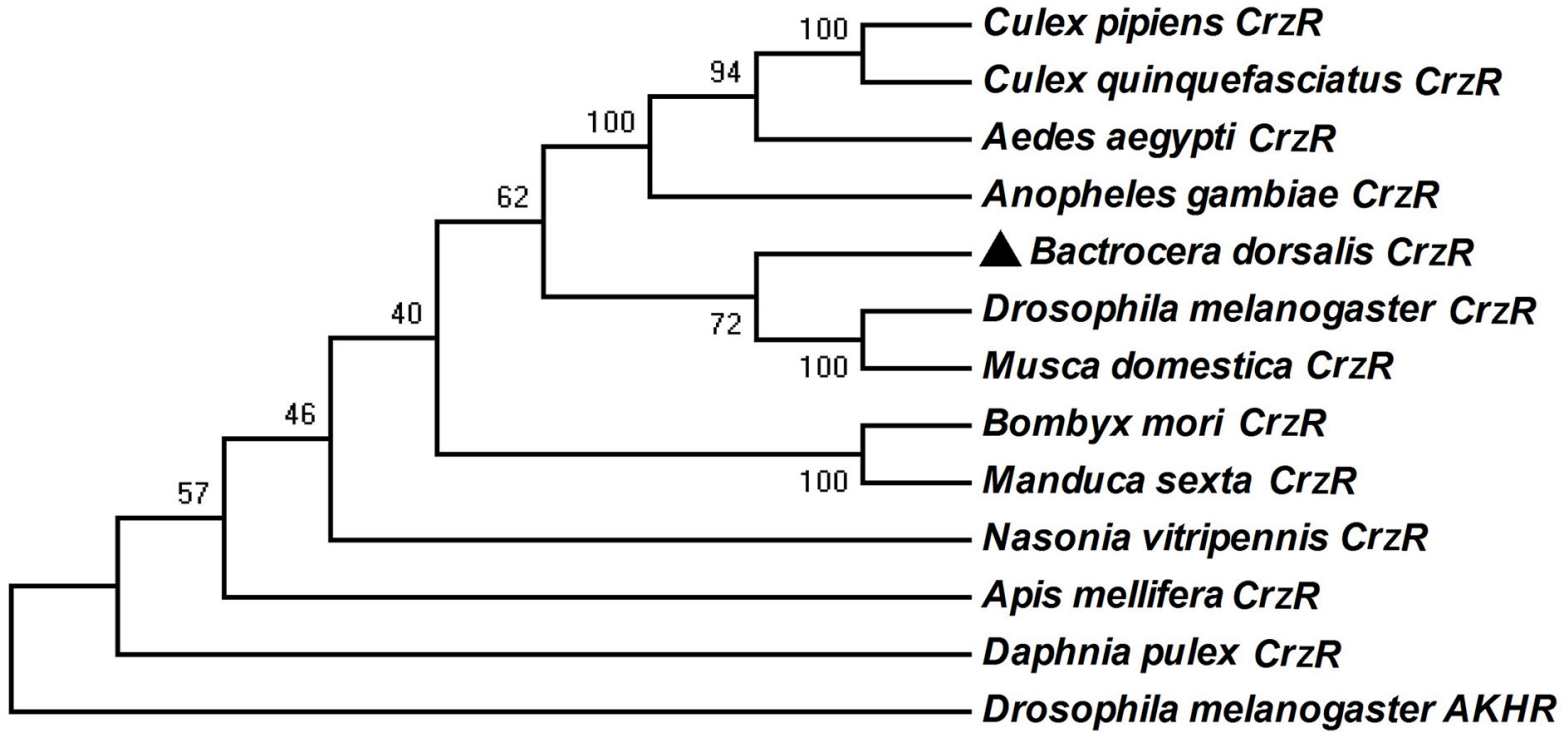
**Phylogenetic analysis of ***BdCrzR*** and its related G protein-coupled receptors**. The tree was constructed with MEGA 6.0 using the neighbor-joining method. Bootstrap support values (1,000 replicates) are indicated on branches. The scale bar represents the number of substitutions per site. *BdCrzR* was assigned with “▲.” GenBank accession numbers and aa sequences of the GPCRs are given in Figure S2.

The *BdCrzR* cDNA (GenBank accession no. KX831394) is composed of an 1827 bp-ORF encoding a putative 608 aa protein. TMHMM server predicted that *BdCrzR* is a typical GPCR with seven transmembrane (TM) domains (Figure S1). Sequencing and BLAST analyses indicated that the *BdCrzR* showed high sequence similarity to the *D. melanogaster* counterpart (Figure 2). Phylogenetic analysis shows that the *BdCrzR* is as closely related to *Musca domestica* as it is to *D. melanogaster* (Figure 3).

### Functional characterization of *BdCrzR*

**Figure 4:**
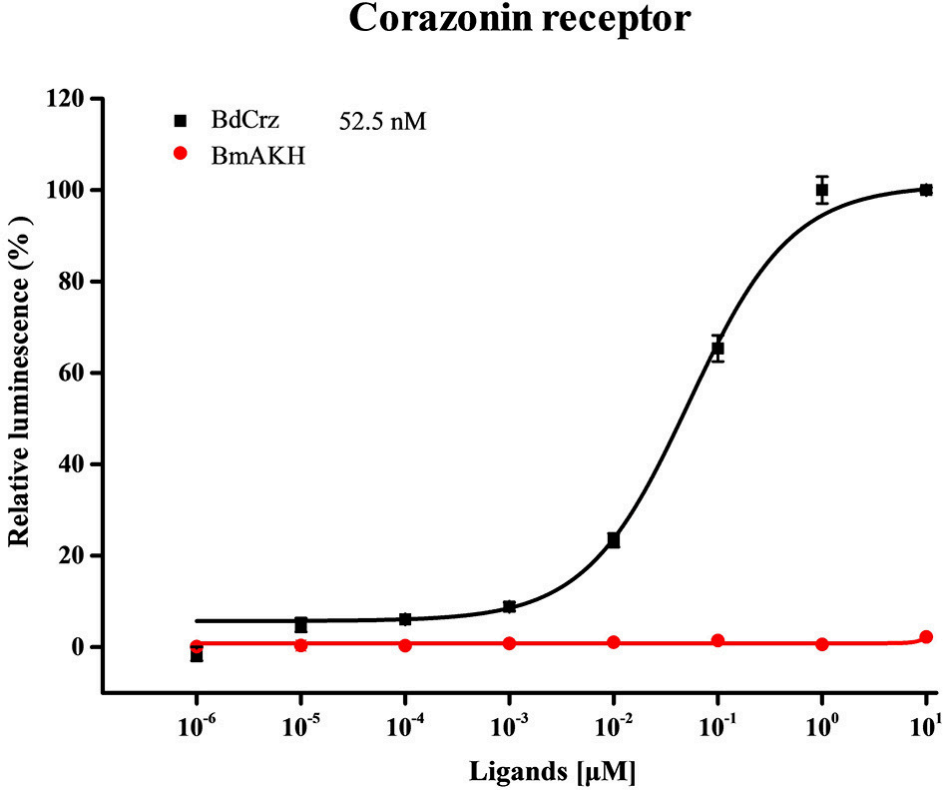
**Concentration-response curves for Ca^**2+**^ responses in ***BdCrzR***-expressing CHO cells**. The sequences of peptides used in this graph are shown in Table 1. Data points represent the mean ± SE based on three independent measurements, each containing three replicates of the tested concentration series. All values were corrected for the negative control (nuclease-free water). The *EC*_50_ values are depicted with their corresponding 95% confidence interval.

The CHO cells transiently expressing *BdCrzR* showed concentration-dependent Ca^2+^ responses when activated with *BdCrz* peptide, but no responses when challenged with *BmAKH* (Table 1). The *EC*_50_ values of the peptides were calculated from concentration-response curves for *BdCrzR*, and the *EC*_50_ value for *BdCrz* was 52.5 nM (Figure 4). CHO cells transfected with empty vector showed no response to any of the peptides tested (data not shown).

### Stage and tissue-specific expression of *BdCrz* and *BdCrzR*

**Figure 5:**
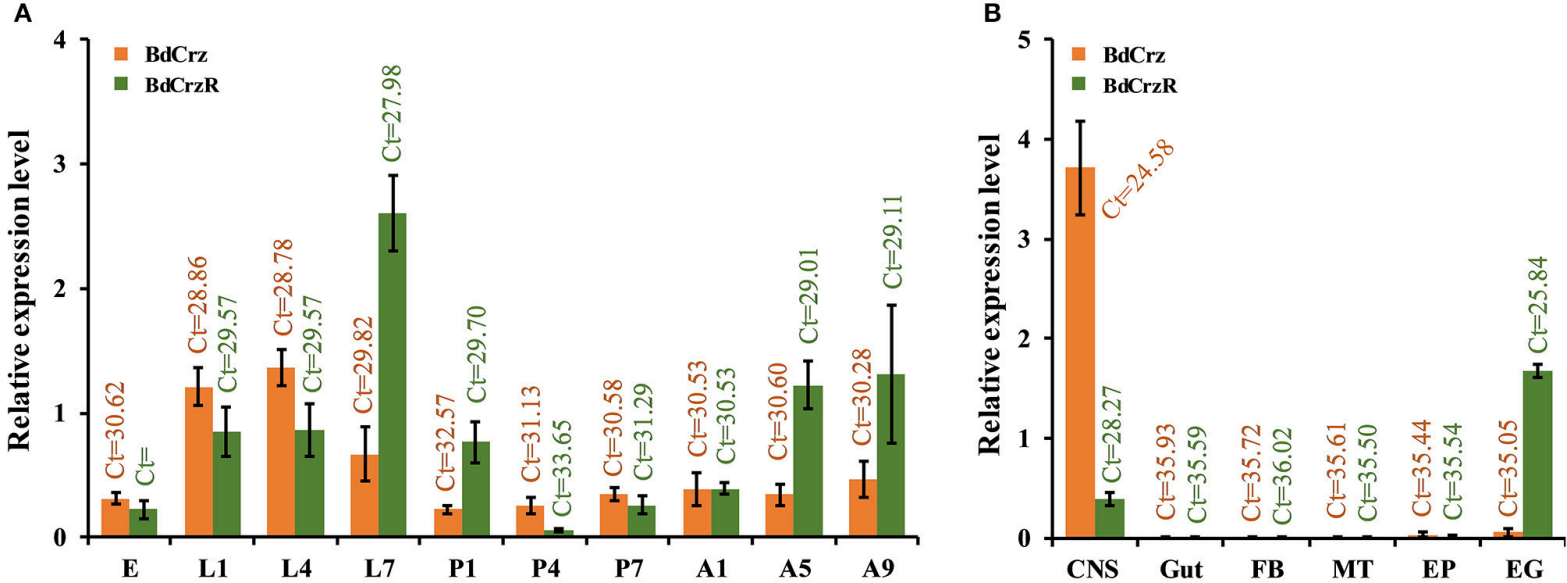
**Relative expression levels of ***BdCrz*** and ***BdCrzR*** in different developmental stages and tissues. (A)** qRCR analysis of the transcription of *BdCrz* and *BdCrzR* in different developmental stages. Expression in adults is from a mixture of females and males. Abbreviations used on the X-axis: E (eggs), L1, L4, L7 (1-, 4-, 7-day-old larvae), P1, P4, P7 (1-, 4-, 7-day-old pupae), A1, A5, A9 (1-, 5-, 9-day-old adults). **(B)** qRCR analysis of the transcription of *BdCrz* and *BdCrzR* in different tissues of 2-day-old 3rd-instar larvae (i.e., 7-day-old larvae, which is the moment prior to larva-pupa transition). Abbreviations used on the X-axis: CNS, central nervous system; Gut, gut; FB, fat body; MT, Malpighian tubules; EP, epidermis; EG, epitracheal gland; containing the Inka cells. The data are presented as mean ± SE based on three biological replicates, all measured in triplicate. Data were normalized using α*-tubulin* as an internal reference gene. Ct, Cycle threshold.

We analyzed the transcriptional levels of the *BdCrz* and *BdCrzR* genes in different developmental stages and in a set of tissues using qPCR (Figure 5). The transcripts of both *BdCrz* and *BdCrzR* were detected in all the developmental stages tested and showed similar expression levels compared to an internal α-tubulin control. Relatively high levels of *BdCrz* were found in the larval stages, low in the egg and pupal stages, and then increased in the adult stage with a medium level. The transcription of *BdCrzR* stayed low in the egg stage, increased in the larval stages, and rose to a peak rapidly in 2-day-old 3rd-instars, which is the moment prior to larval-pupal transition. The peak was followed by a rapid decline on 1-day-old pupae. Relative low levels were obtained during the pupal and adult stages (Figure 5A).

For the tissue-specific expression profiling, we examined the *BdCrz* and *BdCrzR* expression in 2-day-old 3rd-instar larvae. In our experiments, we consider that expression levels with a cycle threshold *Ct* values of 35 or greater are not above the detection limit. Among the six different tissues tested, the *BdCrz* transcript was only detected in the CNS as the *Ct* values for the other tissues was >35. The expression levels of *BdCrzR* mRNA were detected both in the EG, containing the Inka cells, and the CNS tissues. *BdCrzR* showed a relatively higher expression in the EG than CNS, but this result does not necessarily mean that the total expression in the EG tissue is higher than the CNS because the latter is a larger tissue than the EG (Figure 5B).

### Localization of *BdCrz* in the brain

**Figure 6:**
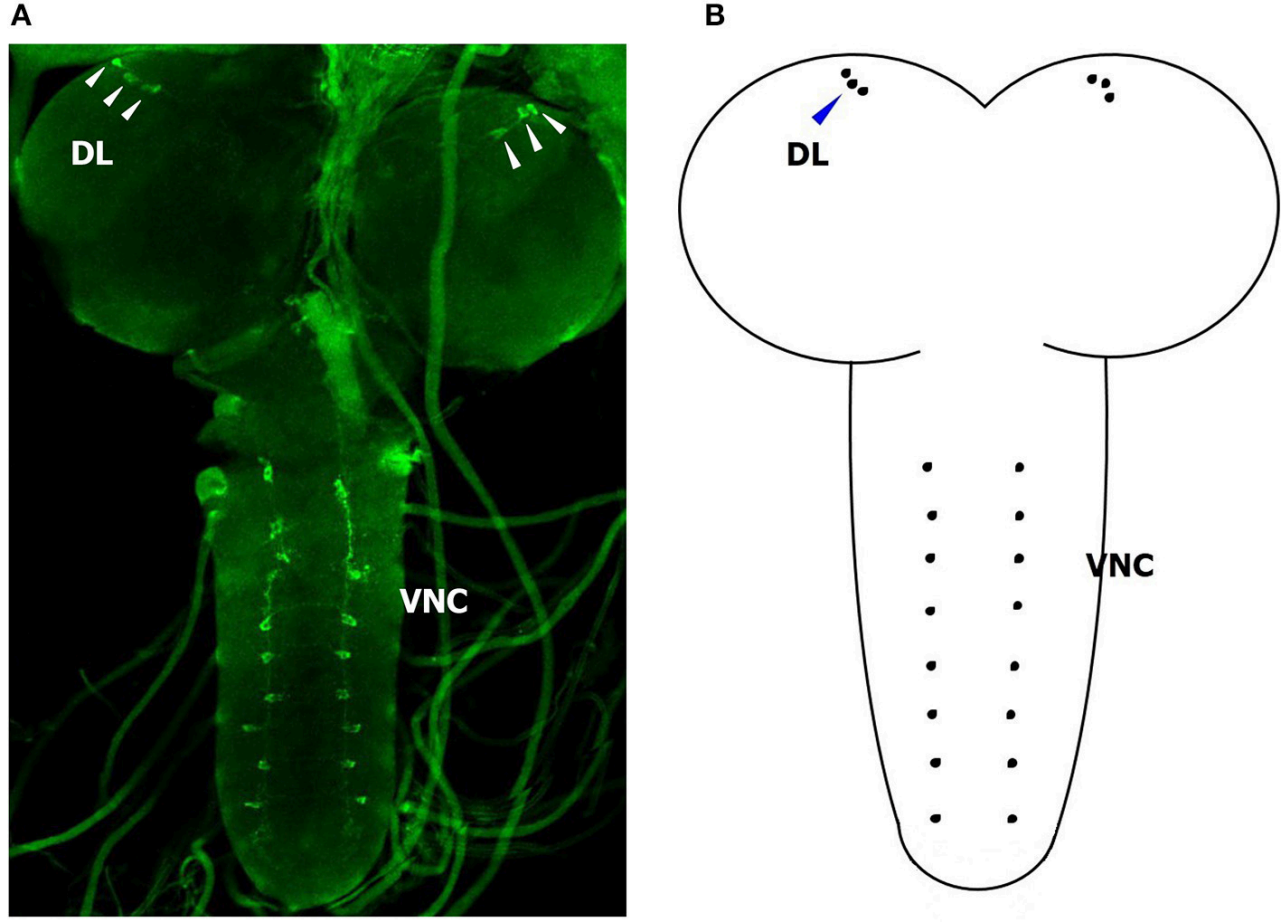
**Immunohistological location of ***BdCrz*** in 2-day-old 3 rd-instar larvae. (A)** The whole brain of a larva stained with Crz-specific antiserum. **(B)** Diagram showing the consolidated view of the immunohistochemistry for *BdCrz* in the larval brain. DL, dorsolateral brain region of the protocerebrum; VNC, ventral nerve cord. Scale bars = 100 μm. Arrows indicate where the neurons are located.

In an attempt to gain insight into the localization of *BdCrz* in the larva, we carried out whole mount immunohistochemistry using a rabbit antibody against *D. melanogaster* mature Crz. Figure 6 shows a representative image of 20 different samples. We found a strong signal in the larval CNS. In each brain lobe of 2-day-old 3rd-instar larvae of *B. dorsalis*, three Crz-immunoreactive neurons were observed in a cluster in the dorsolateral (DL) region of the protocerebrum. In addition, we observed eight pairs of bilateral Crz-immunoreactive neurons in the VNC.

### Effects of RNAi-mediated knockdown of *BdCrzR*

**Figure 7:**
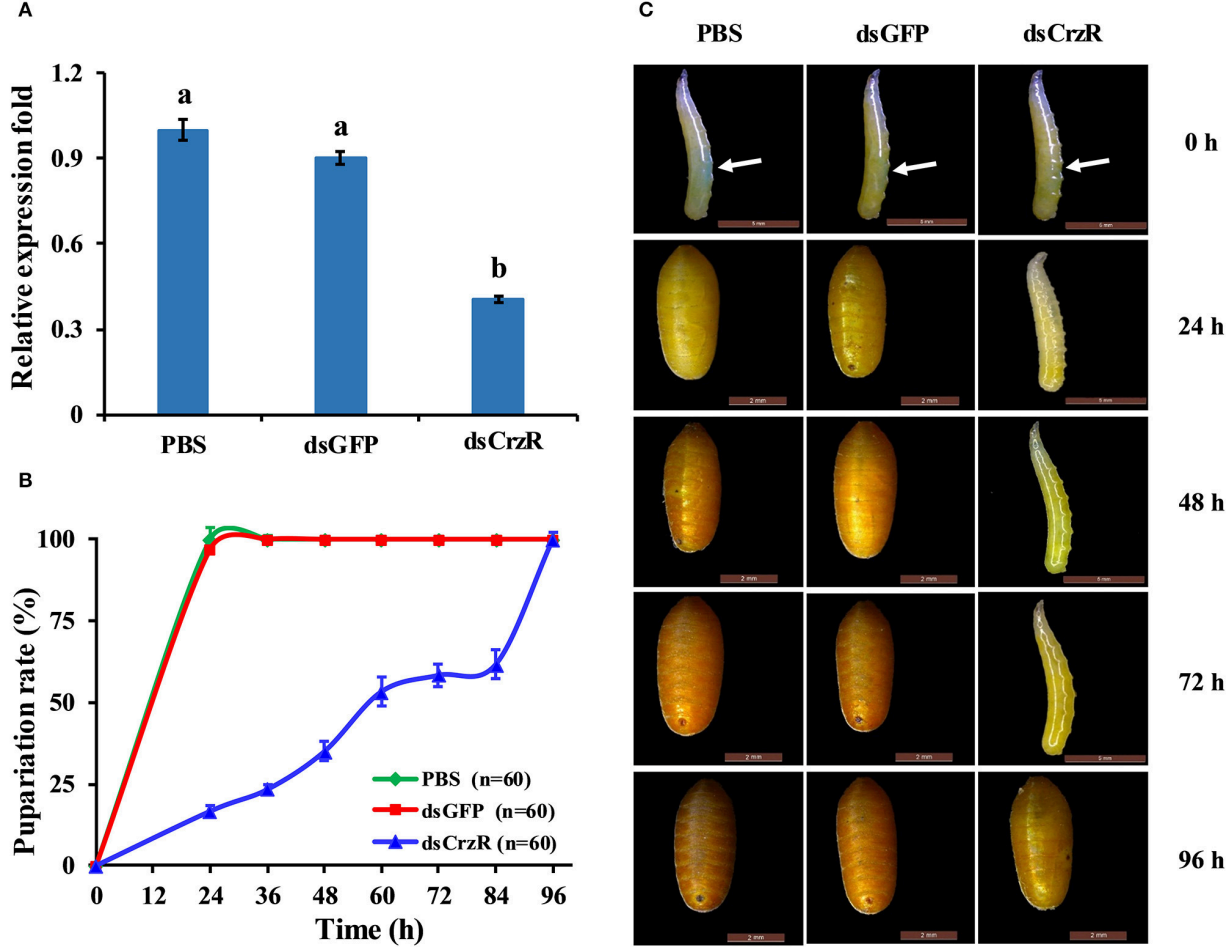
**Effects of ***BdCrzR***-dsRNA injected into 2-day-old 3 rd-instar larvae (i.e., 7-day-old larvae) on the gene transcript levels and development of ***B. dorsalis***. (A)** Relative expression fold of *BdCrzR* in larvae injected with PBS, dsGFP, or dsCrzR. The RNAi efficiency was investigated 24 h after the injection. **(B)** Percentages of larvae that developed into pupae at the representative stages after the injection. The X-axis indicates the time after injection. *n*, the number of insects used. **(C)** Representative phenotypes of the larvae after the injection at different intervals after injection (24, 48, 72, and 96 h). The white arrow indicates where the injection was done. Larvae injected with PBS and dsGFP were used as control. α*-Tubulin* was used as an internal reference gene. Data are presented as means ± SE based on three independent experiments. Gene transcript levels of *BdCrzR* were analyzed using one-way ANOVA, and significant differences between means were tested with a Duncan's Multiple Range Test (*P* = 0.05).

To better understand the function of *BdCrzR* in pupariation, we injected specific dsRNA against *BdCrzR* in 2-day-old 3rd-instar larvae, which is the moment prior to larval-pupal transition, and followed the development daily. At 24 h post-injection, the gene silencing efficacy was 60% compared with the control groups injected with PBS and dsGFP (Figure 7A). Typically, all control larvae had pupated by 24 h post-injection of PBS or dsGFP, while only 17% of dsGFP-injected larvae pupated in this same time frame. When we followed these dsCrzR-injected larvae then all completed the transition into the pupal stage but this was only after 96 h (Figure 7B). Apart from the significant developmental delay there were no abnormalities in morphology and weight in these dsCrzR-injected individuals (Figure 7C, Figure S3). In addition to delayed pupariation, the expression levels of ETH, TH, and DDC also declined about 40–60% in dsCrzR-injected larvae compared to the controls (**Figure 9**).

**Figure 8:**
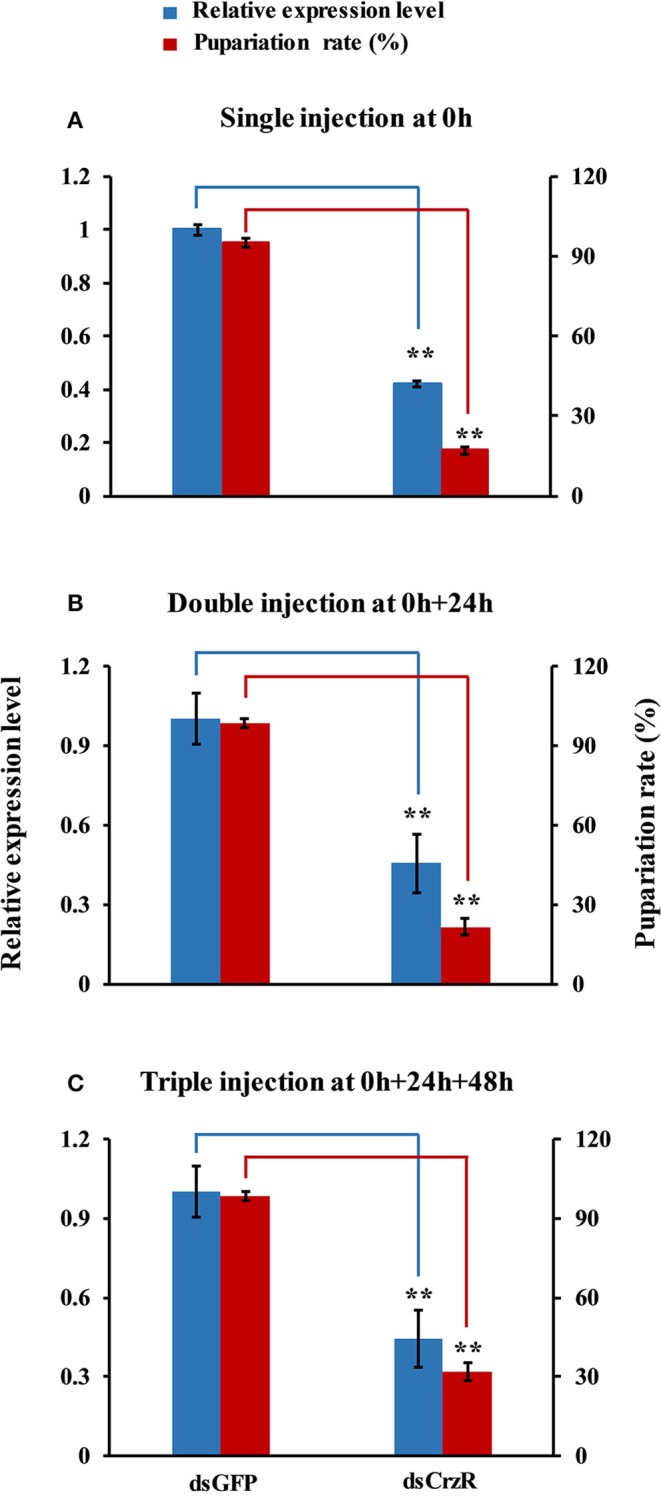
**Effects of a single (A)**, double **(B)**, and triple **(C)** injection of dsCrzR in 2-day-old 3rd-instars on the *BdCrzR* gene transcript levels and development of *B. dorsalis*. Relative expression level of *BdCrzR* gene and the percentage of larvae that had developed into pupae at 24 h after the last injection. In all experiments, the controls were injected with dsGFP, and α*-tubulin* was used as an internal reference gene. Data are presented as means ± SE based on three independent experiments. Data of the single injection come from Figure 7. ^**^P < 0.01, *t*-test.

**Figure 9:**
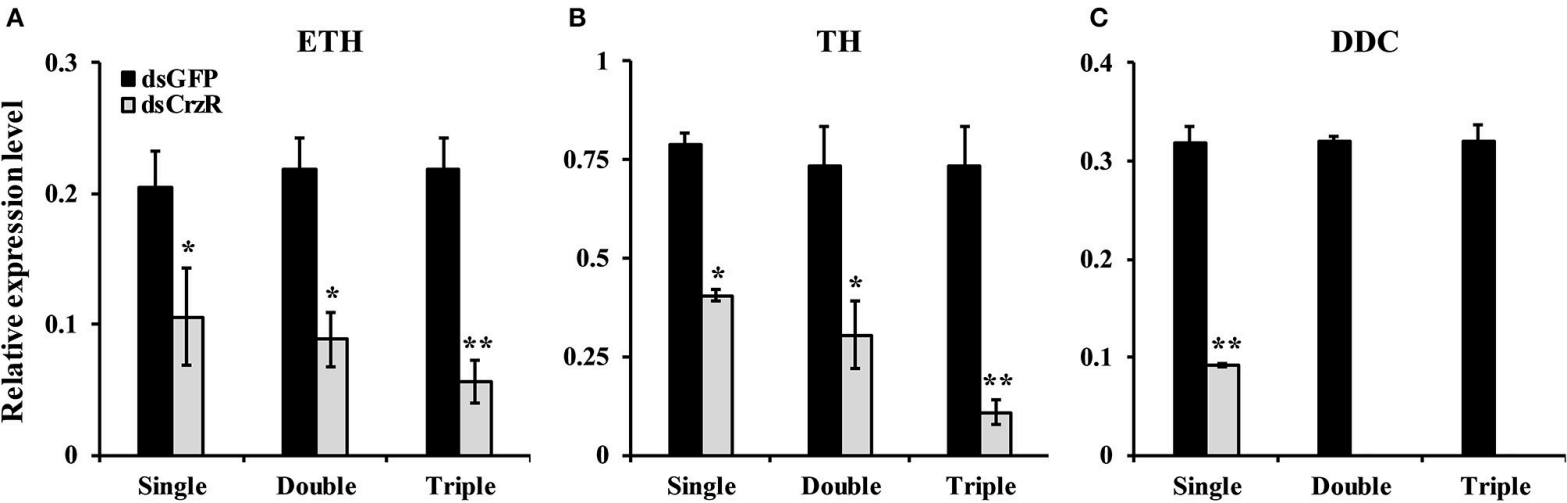
**Effects of single, double and triple silencing of the ***BdCrzR*** gene on the respective expressions of ETH (A)**, TH **(B)**, and DDC **(C)** genes of *B. dorsalis*. Control: dsGFP-injected larvae. The X-axis coordinates indicate the single, double and triple injection of dsRNA. α*-Tubulin* was used as an internal reference gene. Data are presented as means ± SE based on three independent experiments. ^*^P < 0.05, ^**^P < 0.01, *t*-test.

In a second experiment, we injected dsCrzR three times at 0, 24, and 48 h, and compared to control (dsGFP-injected) larvae (because there is no significant difference in PBS- and dsGFP-injection, the next experiments used only dsGFP-injection as control). The percentage of larvae that developed into pupae at 24 h after the double and triple injection was 22 and 32%, respectively; the respective percentage of gene silencing of *BdCrzR* expression was 55 and 56% (Figure 8). In these double and triple injected-larvae with continuous *BdCrzR* expression suppression, the respective expression levels of ETH were reduced by 60 and 75% (Figure 9A), of TH by 55 and 84% (Figure 9B), and of DDC no expression was detected (Figure 9C).

### The rescue of larval-pupal transition by peptide injection in head-ligated larvae

**Figure 10:**
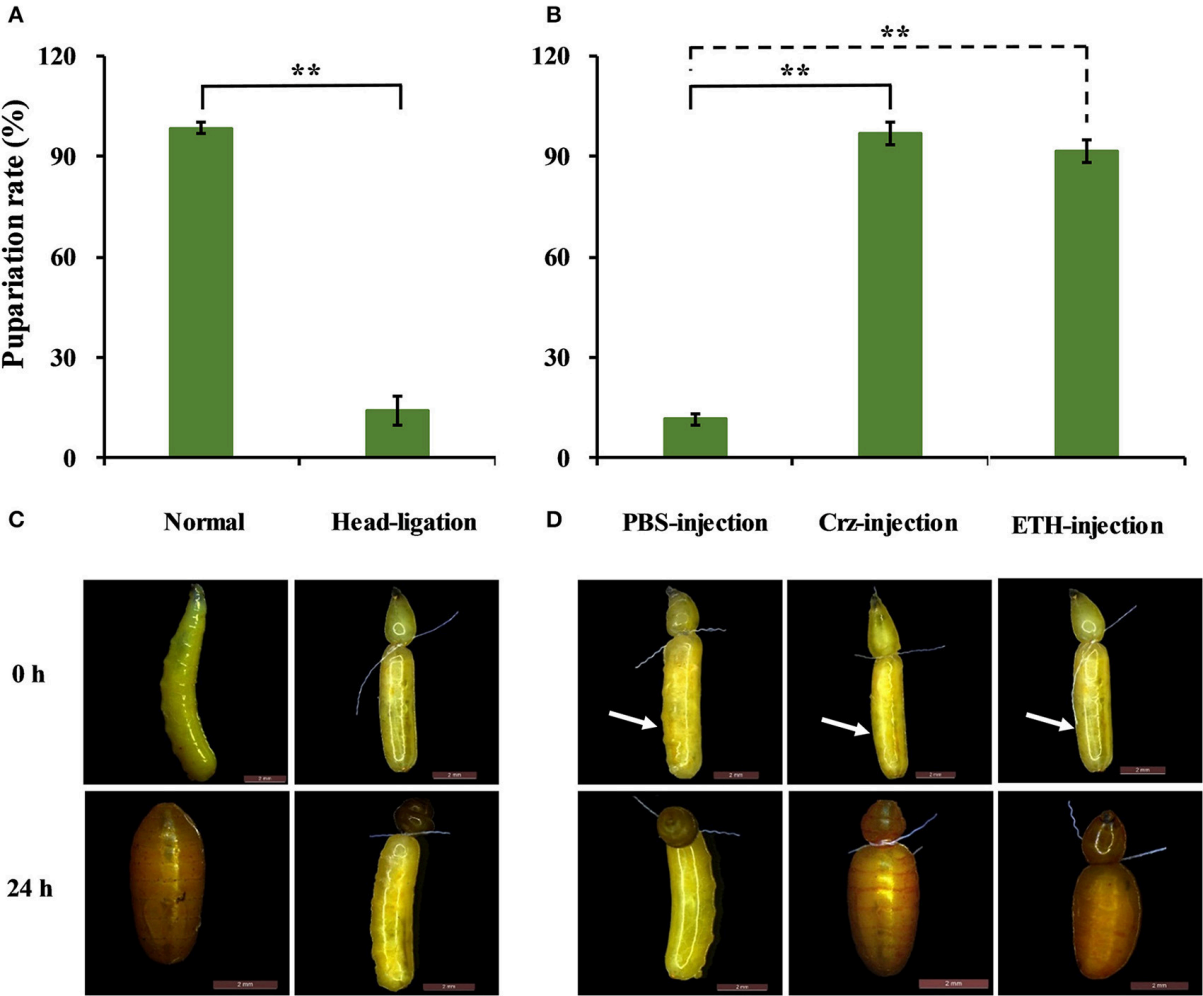
**Effects of Crz and ETH injections into head-ligated 2-day-old 3^**rd**^-instar larvae on larva-pupa transition behavior. (A)** Percentage of head-ligated larvae that developed into pupae at 24 h. Control: Normal 2-day-old 3rd-instar larvae. **(B)** Representative phenotypes of the head-ligated larvae. 2-day-old 3rd-instar larvae were ligated between the first three abdominal segments, and the head-ligated larvae exhibited failure of pupation ecdysis behavior. **(C)** Percentage of 2-day-old 3rd-instar head-ligated larvae that developed into pupae after the injection with PBS, Crz or ETH peptide. **(D)** Representative phenotypes after the injection of Crz or ETH into the head-ligated 2-day-old 3rd-instar larvae. The white arrow indicates where the injection was done. Data are presented as means ± SE based on three independent experiments. ^**^P < 0.01, *t*-test.

Based on the results that Crz is exclusively expressed in the CNS in the brain, we performed the head-ligation in 2-day-old 3rd-instar larvae with the aim to stop the transportation of Crz from the head to the rest of the body. As show in Figure 10A, the transition to the pupal stage in the head-ligated larvae was reduced by 86 to only 14% compared to 100% pupariation in the non-head-ligated larvae (normal larvae) group. In the head-ligated larvae only the head showed the pupal character, while the thorax and abdomen remained in the larval stage (Figure 10B).

To confirm the impact of the CrzR signaling pathway in pupariation, we injected Crz and ETH in head-ligated 2-day-old 3rd-instar larvae to rescue the pupariation behavior. After the injection of Crz, all of the head-ligated larvae made the transition from the larval to the pupal stage (97%). In great contrast, the PBS-injected head-ligated larvae remained in the larval stage, and the same result was also observed with the injection of ETH (Figures 10C,D).

## Discussion

Crz has initially been discovered in *P. americana* as a cardioacceleratory peptide by Veenstra (1989). Upon the reported effects of Crz, much of the evidence supports the hypothesis that the evolutionary ancient function of Crz may have been “to prepare animals for coping with the environmental stressors of the day” (Veenstra, 2009; Boerjan et al., 2010; Kubrak et al., 2016). In the present study, we investigated the molecular characterizations of Crz and its receptor CrzR from *B. dorsalis* and analyzed their potential physiological role in the larval-pupal transition and pupariation behavior.

Crz was for quite some time considered to be highly sequence-conserved, but the sequence variations and the newer data show that this is not really true. As shown in Table 2, Crz has different homologous peptides among different insect species. The *BdCrz* precursor sequence is similar to other CRZ transcripts that have been investigated so far, such as the dipterans and other Crz family members. In the present study, the [His^7^, Ser^11^]-Crz isoform was predicted from *B. dorsalis* that has never been reported before and that is different from the [Arg^7^]-Crz gene that has been characterized in several *Drosophila* species (Choi et al., 2005) and mosquitoes (Hillyer et al., 2012).

**Table 2:** **The different isoforms of corazonin as known to date**.

**Isoform**	**Sequence**
[Arg^7^]-Crz	pGln-Thr-Phe-Gln-Tyr-Ser-Arg-Gly-Trp-Thr-Asn-NH2
[His^7^]-Crz	pGln-Thr-Phe-Gln-Tyr-Ser-His-Gly-Trp-Thr-Asn-NH2
[Thr^4^, His^7^]-Crz	pGln-Thr-Phe-Thr-Tyr-Ser-His-Gly-Trp-Thr-Asn-NH2
[Tyr^3^, Gln^7^, Gln^10^]-Crz	pGln-Thr-Tyr-Gln-Tyr-Ser-Gln-Gly-Trp-Gln-Asn-NH2
[His^4^, Gln^7^]-Crz	pGln-Thr-Phe-His-Tyr-Ser-Gln-Gly-Trp-Thr-Asn-NH2
[Gln^10^]-Crz	pGln-Thr-Phe-Gln-Tyr-Ser-Arg-Gly-Trp-Gln-Asn-NH2
[Met^2^, Arg^10^]-Crz	pGln-Met-Phe-Gln-Tyr-Ser-Arg-Gly-Trp-Arg-Asn-NH2
[His^7^, Ser^11^]-Crz	pGln-Thr-Phe-Gln-Tyr-Ser-His-Gly-Trp-Thr-Ser-NH2

Structural divergence of the Crz genes might have accompanied differential cis-regulatory sequences that result in their distinct expression patterns (Choi et al., 2005). In this study, we investigated the specific presence of *BdCrz*. Similar to an earlier study in *A. gambiae* (Hillyer et al., 2012), the expression levels of *BdCrz* in all developmental stages showed that it was mainly expressed in the larval stage, suggesting that the expression of *BdCrz* might be related to larval development in *B. dorsalis*. The tissue distribution of *BdCrz* is consistent with its proposed role in the larval-pupal transition. Among the tissues tested, we observed high expression levels of *BdCrz* in the CNS. Subcellular localizations of *BdCrz* by immunohistochemistry produced signals in the DL and VNC regions of the larval brain, which seems to be equivalent to the data obtained in other insect species such as *D. melanogaster* (Choi et al., 2005; Lee et al., 2008), *M. domestica* (Sha et al., 2012), *A. gambiae* (Hillyer et al., 2012), and *Phormia terraenovae* (Cantera et al., 1994). These results suggest that the expression of *BdCrz* shows an obvious tissue specificity, and we speculate that the Crz neurons may play an important role in the larval stage in *B. dorsalis*.

The CrzR is a member of the GPCR family having seven TM domains. Since the first CrzR was identified in *Drosophila*, homologous receptors have been predicted in several genome databases (Sha et al., 2012). Here, *BdCrzR* showed a high sequence similarity to *DmCrzR*. Functional analysis showed that *BdCrzR*-transfected CHO cells could be activated by [His^7^, Ser^11^]-Crz with an *EC*_50_ value of 52.5 nM, but did not respond to AKH peptide. Similar reports showed that no effects were found for other peptides with the exception of *DmCrz* on *DmCrzR*-transfected CHO and *Xenopus* oocyte cells, which gave a respective EC_50_ of 18 nM and 1 nM (Cazzamali et al., 2002; Park et al., 2002b). Assays using the CHO and *Xenopus* oocyte-expression system showed that *MsCrzR* has a respective *EC*_50_ value of 200 pM and 75 pM for Crz, and both were insensitive to other peptides assayed up to 1 μM (Kim et al., 2004). The strong conservation of the primary structure and the pharmacological profile between *Drosophila* and *B. dorsalis* CrzRs support their roles as authentic receptors for Crz. However, our result showed a relatively high concentration in cell-based assay with Crz being active at concentrations of nM. During GPCR evolution, the seven TM core structure remained highly conserved, but the regions related to binding and responding to ligands have diverged in size and chemical and physical properties (Römpler et al., 2007; Sha et al., 2012). Thus, it will be interesting to determine whether the binding specific region of *BdCrzR* may have differentiated or the affinity to the ligand decreased during evolution.

Analysis of the developmental stage-specific expression of *BdCrzR* showed higher mRNA levels in the larval stages over the pupal and adult ones. In addition, the produced spikes of CrzR expression occurred during the wandering larval stage (i.e., 2-day old 3rd-instar larvae) just prior to pupal transition. This suggests a role of *BdCrzR* in the regulation of the larval-pupal transition, which coincides with pupariation and/or cuticular melanization (Verleyen et al., 2004; Zdárek et al., 2004; Nachman et al., 2006). Tissue-specific expression analysis of *BdCrzR* revealed that mRNA accumulations were greatest in the EG, which contains the Inka cells, and in the CNS. This is in agreement with studies conducted on moths, whereby northern analysis detected high transcript levels for *MsCrzR* in Inka cells and the CNS (Kim et al., 2004). Specific expression of the CrzR in Inka cells suggested that it may mediate release of ETH. These spatial expression patterns of CrzR reflect peripheral and possible central roles for Crz, similar to those described for eclosion hormone (EH) (Horodyski et al., 1993). Occurrence of CrzR in Inka cells is of considerable functional significance and supports a role for circulating Crz in the regulation of the pupariation behavior (Kim et al., 2004). In addition, the abundant presence of *BdCrzR*-mRNA in the CNS is consistent with the Crz immunolocalization in the CNS. It is likely that centrally released Crz acts on central Crz-expressing neurons, where it could bind to the receptor, and then modulate the activity of the central neurons during the pupariation process.

We knocked down the expression of CrzR via RNAi to investigate its biological role in the larval-pupal transition and pupariation. A single injection of dsCrzR partially silenced CrzR in 2-day-old 3rd-instar larvae and significantly delayed pupariation, indicating that CrzR functions as a major player during the larval-pupal transition and pupariation behavior. This important role was further confirmed by double and triple injections of dsCrzR. In *D. melanogaster*, some reports have shown that peptidergic neurons expressing Crz undergo programmed cell death following eclosion (Choi et al., 2006; Lee et al., 2008). The studies in moths, *M. sexta* (Predel et al., 1999) and *B. mori* (Tanaka et al., 2002), also suggested that there is a functional connection between pupariation and the Crz signaling pathway. This way, a tanning or similar functions of Crz signaling system was implied. In support of this idea, we found significantly reduced transcript levels of TH and DDC, the two enzymes required for dopamine synthesis, were also significantly downregulated in response to RNAi-mediated gene silencing of CrzR. Dopamine is a key molecule in the wandering stage, playing a role as a neurotransmitter (Li et al., 2016). Dopamine is synthesized from L-dopa under the catalysis of DDC, and L-dopa is synthesized from tyrosine by TH. Both DDC and TH participate in the production of dopamine that is required for both cuticle tanning and immune-associated melanization. The amount of TH in the integument was correlated with the degree of cuticle tanning (Huang et al., 2005; Gorman et al., 2007). Based on this evidence, we speculate CrzR-silencing blocks dopamine synthesis, resulting in the inhibition of pupariation and cuticular melanization.

Subsequently, we investigated this hypothesis by the injection of Crz or ETH into head-ligated 2-day-old 3rd-instar larvae just prior to larval-pupal transition. Our data show that head ligation effectively arrested larval-pupal transition, which is most likely due to a blockage of the transportation of pupation-related hormones from head to the thorax and abdomen. Interestingly, the injection of ETH into the abdomen could rescue the pupariation. In the moths, the activation of CrzR in the Inka cell by the injection of Crz could have lead to release ETH, which in turn triggered the transition from preecdysis to ecdysis (Kim et al., 2004). As we know, ETH has shown to be functional exclusively during ecdysis, which happens later than pupariation. It would be possible that is the off-target effects by the injection of a high dose of ETH ending PRLamide with pyrokinin which has been identified as the pupariation factor in flies (Verleyen et al., 2004; Zdárek et al., 2004; Nachman et al., 2006). Therefore, we believe that Crz has no effect on ecdysis timing in *B. dorsalis* rather than pupariation.

In conclusion, we have identified and described the molecular characteristics of [His^7^, Ser^11^]-Crz in *B. dorsalis*, and characterized the molecular and pharmacological properties of its receptor *BdCrzR*. This is the first report, in Diptera as far as we know, to determine the likely role of *BdCrzR* in the larval-pupal transition and pupariation, and we believe this happens via the regulation of hormone secretions by the Inka cells. Therefore, our findings provide further insights into the machinery of insect pupariation, and indicate that the Crz signaling pathway could be a successful new target for the control of important pest insects as *B. dorsalis*.

## Author contributions

HJ, QH, DW, and GS conceived the study and participated in its design. HL helped to perform the GPCR assay. QH performed all of the experiments with the help of SG and EC. JW and GS. provided the materials. QH, HJ, and GS analyzed the data. QH, HJ, JW, and GS wrote the paper.

## Funding

This study was supported in part by the Foundation Project of Southwest University (SWU114049), National Key Research and Development Program (2016YFC1200600), and National Nature Science Foundation of China (31572016).

### Conflict of interest statement

The authors declare that the research was conducted in the absence of any commercial or financial relationships that could be construed as a potential conflict of interest.

## Supplementary material

Figure S1 **cDNA and deduced amino acid residues of the *BdCrzR***. Transmembrane helices are indicated by TM I-TM VII.

Figure S2 **Amino acid sequences and GenBank accession numbers of the CrzR used in the phylogenetic tree analysis**.

Figure S3 **Pupal weight after the injection of *BdCrzR*-dsRNA into 2-day-old 3rd-instar larvae**. Larvae injected with PBS and dsGFP were used as control. Data are presented as means ± SE based on three independent experiments. Data were analyzed using one-way ANOVA, and significant differences between means were tested with Duncan's Multiple Range Test (*P* = 0.05).

Table S1 **Primer sequences used in this study**.
