# Supplementary material for: A Role of Corazonin Receptor in Larval-Pupal Transition and Pupariation in the Oriental Fruit Fly Bactrocera dorsalis (Hendel) (Diptera: Tephritidae)
Source: Front Physiol. 2017 Feb 15;8:77. doi: 10.3389/fphys.2017.00077 (PMC5309247; doi:10.3389/fphys.2017.00077)
Supplement: Figure S1 — cDNA and deduced amino acid residues of the BdCrzR. Transmembrane helices are indicated by TM I-TM VII. [file Image1.PDF]

```
                                                                              tacaaatttacctcgaaaaatcactaa
1    ATGGAAGGTGCAAGTGTGGTGGCAACAACCATTCTCAATCCTCAAGCAGCCGTGGGAGCAATGCTTGAACATTTCGCAGAGAATTTAAGC
1     M  E  G  A  S  V  V  A  T  T  I  L  N  P  Q  A  A  V  G  A  M  L  E  H  F  A  E  N  L  S

91   CACCCACAGCAGTATTACGAGAGTCATGAACCCCATGCACGATTGGCGGCAGCCGCAATTGCCACAACAACACTACCAAATATAGCACTG
31    H  P  Q  Q  Y  Y  E  S  H  E  P  H  A  R  L  A  A  A  A  I  A  T  T  T  L  P  N  I  A  L

181  TCACGTTTGGCGCAAGTTCTCACAATAACACAAAATTTAACGGATAATACCACAATATATCCTTATCTGCCAGACACGTTAGACACAACA
61    S  R  L  A  Q  V  L  T  I  T  Q  N  L  T  D  N  T  T  I  Y  P  Y  L  P  D  T  L  D  T  T

271  CTTACACCGTCAGCAACGAGTAAAGAAGTTTTCTACACTCATGCGCCACAATTCTCCACCACCACGCTGATAAAGGTCTGTGTGCTGGGT
91    L  T  P  S  A  T  S  K  E  V  F  Y  T  H  A  P  Q  F  S  T  T  T  L  I  K  V  C  V  L  G

361  GTGATGGCGATTTTCTCACTCTTCGGCAATATGCTCACCATGTGGAACATCTATAAGACACGCTTCAAGCGGCGTAGCTTGCGCAACTCT
121   V  M  A  I  F  S  L  F  G  N  M  L  T  M  W  N  I  Y  K  T  R  F  K  R  R  S  L  R  N  S
              TM I
451  TGGAATGCCATCTATTCGTTGTTGTTTCATCTATCGATCGCCGATTTGCTTGTAACTGGTTTCTGTATTATCGGCGAGGCGGCCTGGGCG
151   W  N  A  I  Y  S  L  L  F  H  L  S  I  A  D  L  L  V  T  G  F  C  I  I  G  E  A  A  W  A
                                       TM II
541  TACACGGTGCAATGGCGTGGCGGTGATCTTTTGTGCAAATTCTTCAAGCTCTTCCAGATGTTCAGTTTATATTTATCCACTTATGTGATG
181   Y  T  V  Q  W  R  G  G  D  L  L  C  K  F  F  K  L  F  Q  M  F  S  L  Y  L  S  T  Y  V  M
                                                                               TM III
631  GTGCTAATCGGTGTGGATCGTTGGTTTGCGGTGAAATTTCCCATGCGATCGTTATATATGACCAAGAGATGCTATCAATTTCTGGGCATT
211   V  L  I  G  V  D  R  W  F  A  V  K  F  P  M  R  S  L  Y  M  T  K  R  C  Y  Q  F  L  G  I

721  GTCTACATGTCTTCGTTCATATTGAGCATACCACAGTTTTTCATATTTCATTTATCGCGCGGCCCGTTCATCGAAGACTTCCATCAATGC
241   V  Y  M  S  S  F  I  L  S  I  P  Q  F  F  I  F  H  L  S  R  G  P  F  I  E  D  F  H  Q  C
              TM IV
811  GTGACGCATGGCACCTACACGGCACCCTGGCAGGAGCAGAGCTACACCACATTCACGCTATTCAGCACATTTCTGATACCCTTTTGTGTG
271   V  T  H  G  T  Y  T  A  P  W  Q  E  Q  S  Y  T  T  F  T  L  F  S  T  F  L  I  P  F  C  V
                                                                             TM V
901  CTCACCGTCACCTACATTTCCACGTTCCGGGCGATTTCACGAAGTGAAAAGATATTTTTGGGTCCGCAACAGGAGCCGCACACGAGCGCC
301   L  T  V  T  Y  I  S  T  F  R  A  I  S  R  S  E  K  I  F  L  G  P  Q  Q  E  P  H  T  S  A

991  AATTTAATGCATACGAATCGACAACGGCTCATACATAAGGCGAAAATGAATTCGCTGCGACTGTCTTTTGTGATAATAATTGCATTTCTC
331   N  L  M  H  T  N  R  Q  R  L  I  H  K  A  K  M  N  S  L  R  L  S  F  V  I  I  I  A  F  L

1081 ATTTGCTGGGCACCCTACTGTACGCTCATGGTGCTGCTACAGTTTGTCGACATCGACGATGCGACCAGCAAAAGATTAATTGATGGCATA
361   I  C  W  A  P  Y  C  T  L  M  V  L  L  Q  F  V  D  I  D  D  A  T  S  K  R  L  I  D  G  I
          TM VI
1171 TTCTTCTTTGGCATGTCCAATAGTCTGGTGAATCCTCTCATATACGGCGCCTTCCATCTGCACACTATAAAGAGTAAATCGAGCGATAAA
391   F  F  F  G  M  S  N  S  L  V  N  P  L  I  Y  G  A  F  H  L  H  T  I  K  S  K  S  S  D  K
                    TM VII
1261 GGCGGGAATGGTGGCTATAGTCTAAATAGAGCCGACTCGCAGCGTAATCCATCCATGCTAACGGCTGTTACACAAATCGACGGCAGCGGA
421   G  G  N  G  G  Y  S  L  N  R  A  D  S  Q  R  N  P  S  M  L  T  A  V  T  Q  I  D  G  S  G

1351 CGCAGCACACGCGTCAACCGACAGCCCAGCTATTATCGCGCCCAACACAATTTCAGCAACTCCAGCAAGGAGCAGGCCAGCCTGCTGCAG
451   R  S  T  R  V  N  R  Q  P  S  Y  Y  R  A  Q  H  N  F  S  N  S  S  K  E  Q  A  S  L  L  Q

1441 ATGACCCCCACCACCGCCATCGTGCATAAATTCAATTCGAACTCTGAACGCAGTTCAGTGGGTTCATCGCCGCAAACCTGCTCCACAAAT
481   M  T  P  T  T  A  I  V  H  K  F  N  S  N  S  E  R  S  S  V  G  S  S  P  Q  T  C  S  T  N

1531 TTTACACGCAGCGAGATGGGCGACCACGAACACGAGAGCGGCTGCGAAGGGAACGGTGGCGGTGGCGCTAAGCGCGATGCAAATTCACAC
511   F  T  R  S  E  M  G  D  H  E  H  E  S  G  C  E  G  N  G  G  G  G  A  K  R  D  A  N  S  H
```

1621 AGCACCACGGTGGTGTATAGCTTTAAGAAGCCAGCCATTTTGCGCGCACAAAGTTTCGAGGCATTAGCAATTGCGCCGCCCAATAATCGT
541 S T T V V Y S F K K P A I L R A Q S F E A L A I A P P N N R

1711 ATGCAAGCGCCGAAAGCTGCGCCACCTGTGTCCGAGAATGGCTTCCATCGGTCGGTGTGTGTGTTGAGCTTGGAAGATTGTAAGATATGC
571 M Q A P K A A P P V S E N G F H R S V C V L S L E D C K I C

1801 GCCGATGAGCATATCTCTAATGTGTAA
601 A D E H I S N V *

gcgagtgtctgtggaggggagtatgtgtgtatgtgggggatttgcatacaaaatacttttcgaaaaagttccttatttaagtaacacatccggtta
tgtgtgcgtgtacataactgcttgtttacgtaagtatatatgtaggtatatatttata
