# Supplementary material for: A Role of Corazonin Receptor in Larval-Pupal Transition and Pupariation in the Oriental Fruit Fly Bactrocera dorsalis (Hendel) (Diptera: Tephritidae)
Source: Front Physiol. 2017 Feb 15;8:77. doi: 10.3389/fphys.2017.00077 (PMC5309247; doi:10.3389/fphys.2017.00077)
Supplement: Figure S2 — Amino acid sequences and GenBank accession numbers of the CrzR used in the phylogenetic tree analysis. [file Image2.PDF]

>*Aedes aegypti CZR* (from WGS, AAGE02020874.1 and AAGE02020871.1)
MATNLSSILFESLYTNAATATAANGSPSDSYTTTDAFIGTQWTPAAALGSLLATERSETSPDGANGTGWNKVPH
QQPLLLVPSYLSTLATMAANIVEANTTTNVTLSYEDCSLLNVTYGTDGLSTSHPWILCLPHAPTLSKTGVIRVVVL
SAMAIVSLLGNIATMWSIRKNRKSRRLARHNWSAIYSLIFHLSIADLLVTVFCIIGEAAWSYTVEWVAGTVACKLF
KLLQMFSLYLSTYVLVLIGVDRWVAVKYPMKSLNTARRCHRFLCGAYSLSFLLSIPQWMIFRVAKGPFLEDFYQC
VTHGFYTDRWQEQLYTTFTLVFMFIIPLLILIGTYLSTFRTISSSEKIFRIETSAVDRSYYRRSDTNRQRLIHKAKMKS
LRISVVIVVVFIVCWTPYYIMMLIFMFLNPTERLGEDLQTGIFFFGMSNSLINPLIYGAFHLIPIRRRRNQYNQHV
REGSVYFQRSSTCNNQNGNHHQQLLQQPASPPQLELHSQHHHHHHSHHHQPVQPRNSYLAANHKMSHSH
SNLAEEISLMALEKDLKSLDENVNQINLKGTSGGKRSLARKFYSLSQLLRRTRPTKL

>*Anopheles gambiae CZR* (AAQ67361.1)
MPPSFNLSTVTYELISGNVSAALENFTASMAGAFPFEDLHDPASSIARNASFTLGLGLANVIQLQQQQQQQL
HHSPHQYHQQVQHQPQPPSTPFANVSTGQNESLANLLLHPGVHQLSSGLVTLVGDIMAQSGDTILPREECDRL
NISYAFENGTALEIPGLSCYEHAPTLSKSGVIRVIVLSAMAIVSLLGNVATMWNIQKNRKSRRVTRHNWSAIYSLI
FHLSIADVLVTGFCLIGEAAWYYTVDWVAGNLFCKLFKLCQMFSLYLSTYVLVLVGVDRWVAVKYPMKSLNTAR
RCHRFLFVAYLLSFLLSTPQWMIFRVAKGPFVEDFYQCVTHGFYTDRWQEQLYTTFTLVFMFIIPLLILIGTYLSTF
MTISSSEKIFRIDTSAVDRTTYYRRSDTNRQRLIHKAKMKSLRISVVIVVAFVVCWTPYYIMMLIFMFLNPTERFG
EDLQSGIFFFGMSNSLINPLIYGAFHLVPIRQRRNQYNQHVREGSVYFQRSSTFNHHNGHQRNLNPHIKFSNSH
SNLPEEISLMSLEKDLRSLDENVNQIHQKGGGGGGGGGGGGGGVGGGIGLSLGGAAGVDGSRRIKRRSFASK
FLSFSRLLQRNHPTKL

>*Apis mellifera CZR* (NP_001137393.1)
MTILNNYTTYMLLACDNLTNFFNHTRDLTLDSRLWNTSELSHPFHSYNLKNITCLEQAPHSNGNTLFKSLILTIM
AVVSILANLATIYSIVRCRRRHHSWSAIYTLILQLAVADLFVSVFCIGGEAMWNYTVEWIWGNVACKLFKFFQVF
SLYLSTFVLVLIGVDRFFAIRYPMKGMNTADRCLKFIVAAWILSFVLSLPQIIIFRVVQGPFVEKFEQCVTYGFYTEP
WQEQLYVSFGLFSMFLLPLGILIATYVFTIITISRSERMFKVKLANNDICHVNGDVNRRKLMYRAKAKSLRISIVIVT
AFIFWWTPYYTMMIIFMFSCPDKHVSDELQNIIFFFGMSNSLVNPLIYGAFHLWPRKRRNFMHREISTTQRRLT
PTSYGSSYRRDSRETRIPILPKN

>*Bombyx mori CZR* (BAG68427.1)
MDNEGNSTILYDANIMYPSELTLRTEFNTDGNNMNVCAIWPIEKCIEILKLNDTKTDDILGRSFIYNDTQLTCLEH
APVLTKTTVIRASVLSAMAFLSFIGNVATIISIRRSKRCRGRARPSWTAIYSLIFQLSIADLLVTIFCIAGEAAWSFAV
QWYAGNIGCKLFKFLQMLALYLSTFVLVLIGVDRWLAVKYPMKSMATATRSGRLVIIAWVLSVILSIPQAVVFRVA
KGPFFEEFHQCVTHGFYTERWQEQAYTTLSLVFMFILPLIILVSTYVSTVRTIAQSEKVFKPEVRRQEKYFTPDMN
RRRLIDRAKMKSLRMSVVIVAAFLIWWTPYYVMMIIFTFLNPDKNQSEELLNGIFFFGMSNSLVNPIIYGAFHL
WPRKKRSYQHSDRESGGHHASILRRGDNNTSSVRLTTIRSLRSSAKYSNGQNISLL

>*Culex pipiens CZR* (from WGS, AAWU01031675.1, AAWU0103176.1, AAWU0103178.1 and AAWU01031683.1)
MAANLSSFLYESFYGGNATVLDNYTSSTPYDTFQLDSGWPLTTPTVSSTFFPSAEEDNGTGLHQLSTYLSANLTGI
VAVYSQEVCQLQNITFTPDGASSSHPGVFCLPHAPKLSRSGVIRVLVLAAMAIVSLLGNIATMWSIQRNRKSRRL
ARHNWSAIYSLIFHLSIADLLVTVFCIIGEATWSYTVAWLAGTVACKLVKLLQMFSLYLSTYVLVLIGVDRWVAVKY
PMKSLNTARRCHRFLLCAYSLSFLLSIPQWMIFRVAKGPFLEDFYQCVTHGFYSERWQEQLYTTFTLVFMFIIPLLI
LIGTYLSTFRTISSSEKIFRIETSAVDRSYYRRSDTNRQRLIHKAKMKSLRISVVIVVVFIVCWTPYYIMMLIFMFLDP

TDGLVEDLQTGIFFFGMSNSLINPLIYGAFHLIPIRRRHNQYKQHVREGSVYFQRSSTCNNQQPNGTSPHQLQP
SPTPVPPLRSPGSVPLGPLVGTPPIGITSKQLAVARSNSNLAEEISLMSLEKDLKSLDENVNQIHQKNSAAASSRRS
LARKFITLTQLLSRTKPTKL

>*Culex quinquefasciatus CZR* (EDS44142.1)
MIFRVAKGPFLEDFYQCVTHGFYSERWQEQLYTTFTLVFMFIIPLLILIGTYLSTFRTISSSEKIFRIETSAVDRSYYRR
SDTNRQRLIHKAKMKSLRISVVIVVVFIVCWTPYYIMMLIFMFLDPTDGLVEDLQTGIFFFGMSNSLINPLIYGAF
HLIPIRRRHNQYKQHVREGSVYFQRSSTCNNQQPNGTSPHQLQPSPTPVPPLRSPGSVPLGPLVGTPPIGITSKQ
LAVARSNSNLAEEISLMSLEKDLKSLDENVNQIHQKNSAAASSRRSLARKFITLTQLLSRTKPTKL

>*Drosophila melanogaster CZR* (AAM21341.1)
MEDEWGSFDRLPSVPSASMDLETENEVVSNWSTLANFTRLVAGAAPEIVNYTLNMIDVGVGMATDISNLSVS
TTPLPAYAISNSSSLAHTNSRHEAPPMAEQVPEHVMDHAPQLSRSGLLKVYVLAVMALFSLLGNLLTIWNIYKTR
ISRRNSRHTWSAIYSLMFHLSIADVLVTWFCIIGEAAWCYTVQWLANELTCKLVKLFQMFSLYLSTYVLVLIGVDR
WIAVKYPMKSLNMAKRCHRLLGGTYILSLVLSLPQFFIFHVARGPFVEEFYQCVTHGFYTADWQEQMYATFTLV
FTFLLPLCILFGTYMSTFRTISSSEKMFQGSKLANYSTAKLPTQTNRQRLIHKAKMKSLRISVVIIIAFLICWTPYYV
MMIMFMFLNPDKRLGDDLQDAIFFFGMSNSLVNPLIYGAFHLCPGKGGKSSGGGGNNNAYSLNRGDSQRTP
SMLTAVTQVDGTGGSSRQMRAFRQQSYYRSSSNGTAGPGAAPFKEQVGLLHVGPGNGTPGGSVSSGATPQLI
RKGSALLARQPSCLREQEHQQRLLLHEKPSTLVLSYDSQRGGVGVGVASGLLDNNERVSSVEQLAMAVAAAAV
AVDAPDTVEAAAATASADADANVAMEDACSCCHCLSRELVRRQLELQVLPVQ

>*Daphnia pulex CZR* (EFX87464.1)
MTVYHINQSDPNILIPYVITSKDFRDNSSLLEEEAPTLTTSASVKATVLCVMAVASLIGNLATLISIAVSKKGTSSSLY
TLLFQLAISDLLVSVWCLSGEAAWTYTVEWKGGQFLCKTFKFSQVLIGFDRLRAIRRPISHRSCLKPICVAWILSAV
LASPQLLIFSVLRGPFRELFYQCVTYGFYTEEWQEQLYTVFSLLTMFVIPLIVLIGCYVCIFCTIAKKERAFVIRAPHP
SMERWPAESAAVSHFRTRDCQQHRQHSPQRHQHTSFNRRRNNIFKRAKMKSLRISIIIVTAFVICWAPYYFMM
ITFIFLNPDDKLGEDMQSAIFFFGMSNSLVNPLIYGAFHLWQPNIGRSRKLPGSFSTAFTDSVRAGLIVGQRRRPA
EPAADNAVEVAFLGGVLISTSEAAGVVGRDRRSFFDGPKKPKFPSIVLVGLVDQPVPTIVGMPQQNPYPGPPPP
SYAPPSYPAPPSYSPPSYPPPSYSPPAYAPPPSPYAPQSYMAPPPPYQPPQYAPAAPAPMHPPQAQQLPAPELQ
QHEQSVPVPAQEDLKPTEADPPAPAAKNVETESSDAAPSTESTNSDGKSE

>*Musca domestica CZR* (AEI91710.1)
MNLNILTPQMERVPAIDEIIHVASGLTTNFTRNVFGIANSTMKLLNSPANDTNSATISPPLSDMSDNSGDTTITT
TTARHWLLHEGYLVNSSYAFLTNLSYTKPHVYVPASTLPAIEDDEIRLEHAPQLSKSAMIKVYVLSLMALFSLLGNV
LTMWNIYKTRMARRNSRHSWSAIYSLIFHLSIADVLVTGFCIIGEAAWCYTVQWLANELTCKLVKLFQMFSLYLS
TYVLVLIGIDRWIAVKYPMKSLNMAKRCYRLLGGTYILSFILSLPQFFIFHVARGPFVEEFYQCVTHGFYTAEWQE
QLYATFTLVFTLLLPLCILFGSYMSTFRTISSSEKMFQGSKLADYTNKPSSKTNRQRLTHKAKMKSLRISVVIIIAFLIC
WTPYNVMMLIFMFWNPDKRFGEDLQSAIFFFGMSNSLVNPLIYGAFHLCPMKRKTSKKGNNNNGNYSLNRG
DSQRTPSMLTAVTQIDCNGRQVRTYRQTSYYRSISTGSNYKENIGLLQTPTNNGGGTPTPGLIRKSASLRSPHPN
QRSLVHTAGSRRNFNQPLRAGVMDDYDNQRQYHNHSSEIILNDNSSNDAGPVVVSEGQAVSIVLSYDNQRG
GVAAMKRSNTGSISPTAAPFTAPKNGYRTANNLSNTLGIGPATYGNCGNDSVSSV

>*Manduca sexta CZR* (AAR14318.1)
MANGGNNTTLYSDLLFTSDPTLHQDFSVDGSIYTNHQVVWPIEKCIDEHLINDSNIDISKMYMYNGSLVSCLEH

APILTKSTVIRASVLSAMAILSFFGNLATIISIQRGKRGRGRARPSWTAIYSLIFQLSIADLLVTIFCIAGEAAWSFTV
QWYAGNIACKIFKFLQMFALYQSTFILVLIGVDRWLAVKYPMKSMATATRSGRLVVIAWVLSVVLSIPQTVVFRV
AKGPFVEEFYQCVTHGFYTERWQEQAYTTLSLVFMFVLPLVILISTYVSTVRTIARSEKVFKPEVRRQEKYFTPDM
NRRRLIDRAKMKSLRMSVVIVAAFLVWWAPYYVMMIIFTFLNPDKDQSEELLSGIFFFGMSNSLVNPVIYGAFH
LWPKKKRSHRHSDRESGGHHASLLRRGDNNTSSIRLTTIRSLRSSAKYSNGHNISLL

>*Nasonia vitripennis CZR* (from WGS, AAZX01006363.1, AAZX01010633.1,AAZX01000283.1, AAZX01013590.1 and AZX01015155.1)
MYVLEEEPCLNIRNASGLISTNILRNSSCLGHAPQLTYGAYLRAVVLLSMTLLSFLANLATIWSIKSNKRKSQNCSA
IYSLILHLSVADLFVTVFCMGGEALWSYNVAWIWGNTACKAFKFLQMFSLYLSTFVLVLIGIDRFVAVKYPMKTL
NTAKKCNQLISFIWFISFILSTPQVVIFHVAQGPFIEDFSQCVTHGFYTEVWQEQLYTTLSLIFMFIMPLTILITTYM
STVITIARSERLFKSELANSSSAHKTGDVNRRRLIHRAKTKSLRISVVIVVAFVLWWTPYYIMMIIFMFLNPDKHV
SADMQKGIFFFGMSNSLVNPLIYGAFHLWPQKKNRKHRENSIMQLRSTTTNASLMMDHRSTNARFTKQTKYS
HIQTHEGLSVNHDTVVVHLIENSEKNKFCGRAAKIILHYNLPTKSN

>*Drosophila melanogaster AKHR* (AAC61523.1)
MAKVAEENDHRDLSNWSNVNDTNGTIHLTKDMVFNDGHRLSITVYSILFVISTIGNSTVLYLLTKRRLRGPLRIDI
MLMHLAIADLMVTLLLMPMEIVWAWTVQWLSTDLMCRLMSFFRVFGLYLSSYVMVCISLDRYFAILKPLKRSY
NRGRIMLACAWLGSVVCSIPQAFLFHLEEHPAVTGYFQCVIFNSFRSDFDEKLYQAASMCSMYAFPLIMFIYCYG
AIYLEIYRKSQRVLKDVIAERFRRSNDDVLSRAKKRTLKMTITIVIVFIICWTPYYTISMWYWLDKHSAGKINPLLR
KALFIFASTNSCMNPLVYGLYNIRGRMNNNNPSVNNRHTSLSNRLDSSNQLMQKQLTNNSLLNGRGQVMAA
AVSATTKLANVVSLKGTANGNGSAAAAGTVPITPPLTVTIAPLATDDEANDDSCLSAVTIRCQDQSPIRQK
