# Supplementary figures and images for: A Role of Corazonin Receptor in Larval-Pupal Transition and Pupariation in the Oriental Fruit Fly Bactrocera dorsalis (Hendel) (Diptera: Tephritidae)
Source: Front Physiol. 2017 Feb 15;8:77. doi: 10.3389/fphys.2017.00077 (PMC5309247; doi:10.3389/fphys.2017.00077)

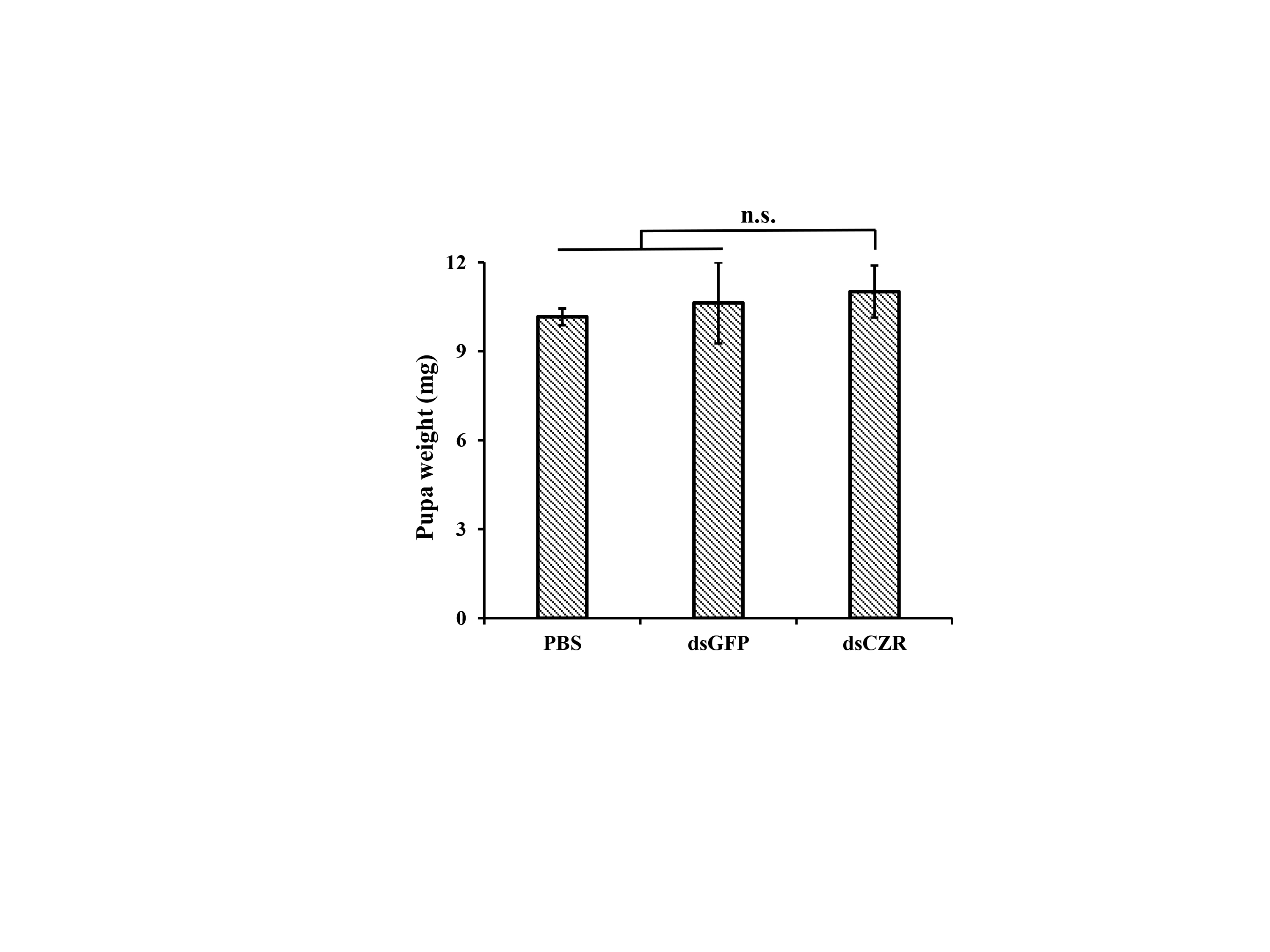

Supplement: Figure S3 — Pupal weight after the injection of BdCrzR-dsRNA into 2-day-old 3rd-instar larvae. Larvae injected with PBS and dsGFP were used as control. Data are presented as means ± SE based on three independent experiments. Data were analyzed using one-way ANOVA, and significant differences between means were tested with Duncan's Multiple Range Test (P = 0.05). [file Image3.JPEG]
