# Supplementary material for: A Role of Corazonin Receptor in Larval-Pupal Transition and Pupariation in the Oriental Fruit Fly Bactrocera dorsalis (Hendel) (Diptera: Tephritidae)
Source: Front Physiol. 2017 Feb 15;8:77. doi: 10.3389/fphys.2017.00077 (PMC5309247; doi:10.3389/fphys.2017.00077)
Supplement: Table S1 — Primer sequences used in this study. [file Table1.pdf]

Table S1. Primer sequences used in this study

Target	Direction	Sequence 5' to 3'
BdCrz (the first-round)	Forward	CAAACGGCTTTTTATTAAAAC
	Reverse	CTACTAAATTCGTGTGGAGTC
BdCrz (the second-round)	Forward	CATCATGTTCAAATTATTCTTC
	Reverse	GCCATAATCATGTTTTAATGC
BdCrzR (the first-round)	Forward	ACCTCGAAAAATCACTAAATGG
	Reverse	CACATACTCCCCTCCACAGAC
BdCrzR (the second-round)	Forward	CTAAATGGAAGGTGCAAGTGTG
	Reverse	CTCGCTTACACATTAGAGATATGC
BdCrz (qRCR)	Forward	TTGCCGAAATGCTCCAACAA
	Reverse	CCATAATCGTTCGTCTCGGC
BdCrzR (qPCR)	Forward	TGCTCACCGTCACCTACATT
	Reverse	TCACAAAAGACAGTCGCAGC
BdETH (qPCR)	Forward	CGTAAACCGGATCAACGACT
	Reverse	GCGCATAAAGTCTCCACCAT
BdTH (qPCR)	Forward	GCCGAACATAACATCAGCGT
	Reverse	CTCATCGCGCAATTTCTGGA
BdCCD (qPCR)	Forward	AGCCAACTCATATCCAGCCA
	Reverse	CTAAAGCCTCACCACGAAGC
α-Tubulin (qRCR)	Forward	CGCATTCATGGTTGATAACG
	Reverse	GGGCACCAAGTTAGTCTGGA
BdCrzR (dsRNA)	Forward	taatacgactcactataggTTACACAAATCGACGGCAGC
	Reverse	taatacgactcactataggGCTGTGTGAATTTGCATCGC
GFP (dsRNA)	Forward	taatacgactcactataggCAGTTCTTGTTGAATTAGATG
	Reverse	taatacgactcactataggTTTGGTTTGTCTCCCATGATG
